# Mitochondria-Targeted Delivery of Camptothecin Based on HPMA Copolymer for Metastasis Suppression

**DOI:** 10.3390/pharmaceutics14081534

**Published:** 2022-07-23

**Authors:** Xiaoli Yi, Yue Yan, Xinran Shen, Lian Li, Yuan Huang

**Affiliations:** Key Laboratory of Drug-Targeting and Drug Delivery System of the Education Ministry, Sichuan Engineering Laboratory for Plant-Sourced Drug and Sichuan Research Center for Drug Precision Industrial Technology, West China School of Pharmacy, Sichuan University, No. 17, Block 3, South Renmin Road, Chengdu 610041, China; yixiaoli728@126.com (X.Y.); xinwen93696@126.com (Y.Y.); yy18224019649@163.com (X.S.); liliantripple@163.com (L.L.)

**Keywords:** camptothecin, metastasis, mitochondria, 2-(dimethylamino) ethyl methacrylate

## Abstract

Poor anti-metastasis effects and side-effects remain a challenge for the clinical application of camptothecin (CPT). Mitochondria can be a promising target for the treatment of metastatic tumors due to their vital roles in providing energy supply, upregulating pro-metastatic factors, and controlling cell-death signaling. Thus, selectively delivering CPT to mitochondria appears to be a feasible way of improving the anti-metastasis effect and reducing adverse effects. Here, we established a 2-(dimethylamino) ethyl methacrylate (DEA)-modified *N*-(2-hydroxypropyl) methacrylamide (HPMA) copolymer–CPT conjugate (P-DEA-CPT) to mediate the mitochondrial accumulation of CPT. The mitochondria-targeted P-DEA-CPT could overcome multiple barriers by quickly internalizing into 4T1 cells, then escaping from lysosome, and sufficiently accumulating in mitochondria. Subsequently, P-DEA-CPT greatly damaged mitochondrial function, leading to the reactive oxide species (ROS) elevation, energy depletion, apoptosis amplification, and tumor metastasis suppression. Consequently, P-DEA-CPT successfully inhibited both primary tumor growth and distant metastasis in vivo. Furthermore, our studies revealed that the mechanism underlying the anti-metastasis capacity of P-DEA-CPT was partially via downregulation of various pro-metastatic proteins, such as hypoxia induction factor-1α (HIF-1α), matrix metalloproteinases-2 (MMP-2), and vascular endothelial growth factor (VEGF). This study provided the proof of concept that escorting CPT to mitochondria via a mitochondrial targeting strategy could be a promising approach for anti-metastasis treatment.

## 1. Introduction

CPT is a promising chemotherapeutic agent due to its potent inhibitory effect against DNA topoisomerase [1]. Although targeted delivery of CPT has been reported to improve its cytotoxicity, the anti-metastasis effect is poor [2,3,4]. Furthermore, recent studies disclosed that nuclear DNA damage by CPT could induce a massive release of double-stranded DNA (dsDNA), which could subsequently stimulate a strong immune response to initiate the intestinal diarrhea, a life-threatening side-effect of camptothecin [5,6]. Thus, exploring new strategies to improve its anti-metastasis efficacy while reducing its side-effects is needed.

Due to the imperative roles in regulating cellular metabolism and cell-death signaling [7,8], mitochondria not only provide various metabolites and antiapoptotic proteins for rapid tumor growth but also facilitate the migration and invasion of tumor cells by offering abundant energy and upregulating pro-metastatic factors [9,10,11]. Increasing evidence has shown that targeted induction of mitochondria dysfunction by therapeutics (i.e., doxorubicin, lonidamine, and metformin) hold tremendous potential for suppressing both primary tumors and metastases [12,13,14]. Notably, CPT can be a cellular respiration inhibitor to impair mitochondrial without causing side-effects such as diarrhea [15,16]. Thus, selectively delivering CPT to mitochondria appears to be a feasible way of improving the anti-metastasis effect and reducing adverse effects.

Free drugs rely on simple diffusion to randomly interact with the organelles of tumor cells [17]. Thus, the amount of CPT that accumulates in mitochondria is very limited due to the multiple intracellular barriers to reaching mitochondria and the extremely poor permeability of the mitochondrial inner membrane, which represent formidable hurdles [18,19]. Recently, various ligands have been reported to improve mitochondrial accumulation, such as triphenylphosphonium bromide, mitochondrial penetration peptide, and mitochondrial targeting sequence [20,21,22]. However, those lipophilic moieties might deteriorate the solubility of the highly hydrophobic CPT. 2-(Dimethylamino) ethyl methacrylate, a hydrophilic small molecular containing a tertiary amino group, might be a promising ligand to facilitate the transport of CPT to mitochondria [23]. Nevertheless, the modification content of DEA for effective mitochondria targeting remains unknown.

**Scheme 1:**
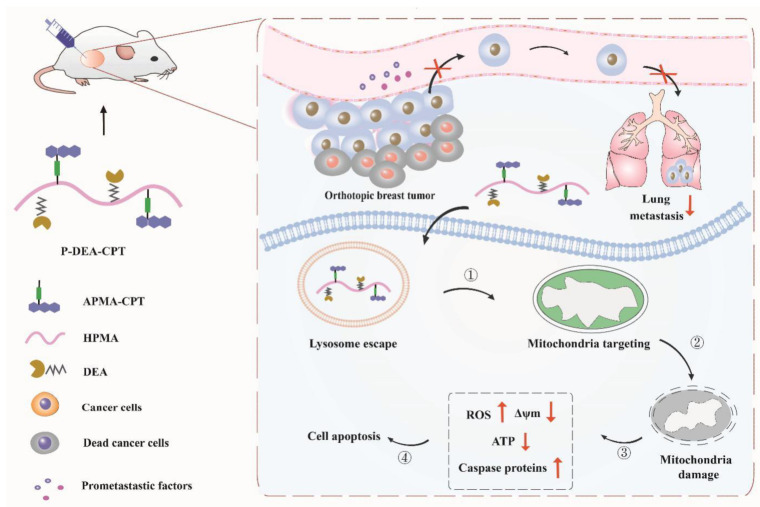
Schematic illustration of P-DEA-CPT for damaging mitochondria and suppressing metastasis. After internalized into cancer cells, P-DEA-CPT ① escaped from lysosome, ② targeted mitochondria, ③ damaged mitochondria and ④ finally induced cell apoptosis.

Herein, we designed a DEA-modified HPMA copolymer–CPT conjugate (P-DEA-CPT) consisting of (1) HPMA copolymer acting as a carrier and (2) DEA acting as the mitochondria-targeting moiety (Scheme 1). DEA was decorated on the side-chain, and its modification degree was screened to ensure sufficient mitochondria accumulation. P-DEA-CPT greatly enhanced mitochondrial location of CPT in cancer cells. Subsequently, P-DEA-CPT induced mitochondrial dysfunction, thereby efficiently curbing the growth and metastasis of breast cancer. Thus, our study unleashed the potential for anti-metastasis treatment of CPT via a mitochondria-targeted delivery system.

## 2. Materials and Methods

### 2.1. Materials

Camptothecin (CPT) (>98%) and 2-(dimethylamino) ethyl methacrylate (DEA) were acquired from Giant Medical Technology Co., Ltd. (Chengdu, China), whereas *N*-(3-aminopropyl) methacrylamide hydrochloride was purchased from Bide Pharmaceutical Technology Co., Ltd. (Shanghai, China). Lyso-tracker Red was provided by Thermo Fisher Scientific (Shanghai, China). MitoTracker Red, Mitochondrial Membrane Potential Assay Kit, Reactive Oxygen Species Assay Kit, ATP Assay Kit, Caspase 3 and Caspase 9 Activity Assay Kit, and Tissue Mitochondria Isolation Kit were all obtained from Beyotime Biotechnology Co., Ltd. (Shanghai, China). All other reagents were of analytical grade or above.

### 2.2. Cells and Animals

Murine breast cancer cells (4T1) and human umbilical vein endothelial cells (HUVECs) were obtained from Icell Biotech Co., Ltd. (Shanghai, China) and the Chinese Academy of Science Cell Bank for Type Culture Collection (Shanghai, China), respectively. Cells were incubated in a homothermal cell incubator (37 °C) with 5% CO_2_. RPMI 1640 medium (Gibco, Invitrogen Co., Grand Island, NY, USA) supplemented with 10% (*v*/*v*) fetal bovine serum (FBS) and 1% (*v*/*v*) antibiotics (penicillin–streptomycin) was used for 4T1 cells, and Dulbecco’s modified Eagle’s medium (Gibco, Invitrogen) supplemented with 10% (*v*/*v*) fetal bovine serum (FBS) and 1% (*v*/*v*) antibiotics (penicillin–streptomycin) was used for HUVECs.

Female BALB/c mice (6 to 10 weeks) were provided by Chengdu Dashuo Experimental Animal Co., Ltd. (Chengdu, China). All animal experiments strictly abided by the Guidelines of Medical Ethics Committee of Sichuan University.

### 2.3. Synthesis, Characterization, and Mitochondria-Targeting Capacity of HPMA Copolymers with Different Modification Ratios of DEA

FITC-labeled HPMA copolymers conjugated with different 2-(dimethylamino) ethyl methacrylate (DEA) ratios were synthesized, and their mitochondrial accumulation was evaluated. Firstly, *N*-(2-hydroxypropyl) methacrylamide (HPMA) and *N*-(3-aminopropyl) methacrylamide–fluorescein isothiocyanate monomer (APMA–FITC) were synthesized in the same way as our previous study [24]. Then, FITC-labeled HPMA polymers with various DEA modification amounts were obtained by direct radical polymerization of monomers. Briefly, the monomers (APMA–FITC:DEA:HPMA = 5:0~13:95~82 mol.%) and azobisisobutyronitrile (2 wt.%) as the initiator were dissolved in dimethyl sulfoxide and stirred for 24 h at 50 °C under argon atmosphere. Products were precipitated into diethyl ether and freeze-dried after being purified by dialysis. The obtained HPMA conjugates modified with 0%, 5%, 10% and 13% molar ratios of DEA were defined as PFITC, P-DEA (5%)-FITC, P-DEA (10%)-FITC, and P-DEA (13%)-FITC, respectively. Next, a Fast Protein Liquid Chromatograph (FPLC, GE Healthcare Life Science, Piscataway, NJ, USA) was used to detect the molecular weight and polydispersity of these copolymers. The zeta potential of the copolymers was estimated by Zetasizer Nano ZS90 at 25 °C (Malvern Instruments, Malvern, UK). The amount of FITC contained in these copolymers was determined via ultraviolet spectroscopy GENESYS 180 (Thermo fisher technologies, South San Francisco, CA, USA).

Then, mitochondria-targeting capacity of various HPMA copolymers was evaluated in 4T1 cells. Briefly, after being incubated with the above-obtained copolymers (FITC dose, 10 μg·mL^−1^) for 4 h, the mitochondria of 4T1 cells were extracted by grinding the cells in mitochondria extraction reagent under ice bath 20 times, and cell debris was removed by centrifuging at 600× *g* for 10 min at 4 °C. Next, the obtained supernatant containing mitochondria was centrifuged at 11,000× *g* for 15 min at 4 °C, and the mitochondria pellets were collected. Finally, the fluorescence intensity of FITC in mitochondria was quantitatively determined via flow cytometry (FACS Calibur, BD, Franklin Lakes, NJ, USA).

Furthermore, the safety of these copolymers was investigated in both 4T1 tumor cells and HUVECs. After seeding in 96-well plates, 4T1 cells and HUVEC were treated with P-FITC, P-DEA (5%)-FITC, P-DEA (10%)-FITC, and P-DEA (13%)-FITC at various predetermined concentrations for 48 h. Then, fresh MTT agent was added and cultured for another 4 h. Finally, the amount of formazan in each well was determined by Varioskan Flash 902-ULTS (Thermo Scientific, Sunnyvale, CA, USA) after being dissolved in 200 μL of dimethyl sulfoxide (DMSO). The relative cell viability was calculated as the absorption value of experimental wells reverse that in the drug-free medium treated group.

### 2.4. Synthesis and Characterization of HPMA Copolymer-CPT Conjugates

The azelaic acid–camptothecin conjugate (LA–CPT) and *N*-(3-aminopropyl) methacrylamide hydrochloride–azelaic acid–camptothecin (APMA–LA–CPT) monomer were synthesized as described in our previous study [23]. Subsequently, CPT-loaded HPMA copolymers with (13% molar ratio, P-DEA-CPT) or without DEA modification (P-CPT) were synthesized according to the same procedure mentioned above. The molecular weight, polydispersity, zeta potential, and CPT loading capacity of P-CPT and P-DEA-CPT were evaluated using the same method in Section 2.3.

### 2.5. Cellular Uptake, Lysosome Escape, and Mitochondrial Targeting of HPMA Copolymer–CPT Conjugates

The 4T1 cells were treated with free CPT, P-CPT and P-DEA-CPT (equivalent CPT dose, 20 μg·mL^−1^) for 4 h. Then, 4T1 cells were harvested, and the fluorescence intensity of CPT was qualitatively observed via a laser scanning confocal microscope (CLSM, Zeiss LSM 800, Oberkochen, Germany) and quantitatively detected via flow cytometry.

Then, whether HPMA copolymer–camptothecin conjugates could escape from lysosome and accumulate in mitochondria was investigated. The 4T1 cells were incubated with P-CPT or P-DEA-CPT (equivalent CPT dose, 20 μg·mL^−1^) for 4 h. After being labeled with Mito-Tracker Red or Lyso-Tracker Red, cells were visualized under CLSM.

### 2.6. In Vitro Mitochondrial Damage by HPMA Copolymer–Camptothecin Conjugates

#### 2.6.1. Reactive Oxygen Species Detection

The level of reactive oxygen species in cancer cells after CPT polymeric conjugate treatment was detected using a DCFH-DA probe. The 4T1 cells were seeded in 12-well plates and then treated with free CPT, P-CPT, and P-DEA-CPT (equivalent CPT dose, 20 μg·mL^−1^) for 8 h. Afterward, cells were harvested and incubated with DCFH-DA solution for 30 min at 37 °C followed by flow cytometry analysis.

#### 2.6.2. Mitochondrial Membrane Potential Detection

Mitochondrial membrane depolarization in 4T1 cells was measured by JC-1 probe, which is a cationic lipophilic dye that can form red fluorescent complexes known as J-aggregates in the mitochondrial matrix under normal mitochondrial membrane potential (Δψm). In contrast, Δψm loss can induce decreased J-aggregates in the mitochondria and increased monomeric form (J-monomer) emitting green fluorescence in the cytosol. Therefore, the red (J-aggregate)/green (J-monomer) fluorescence intensity ratio is a direct evaluation of the Δψm depolarization. The 4T1 cells received the same drug treatments as in ROS detection assay and were stained with JC-1 probe for 30 min at 37 °C in the dark before being measured by flow cytometry.

#### 2.6.3. Measurement of ATP Level

Adenosine 5′-triphosphate (ATP) could offer energy and catalyze luciferase to generate fluorescence. On this basis, intracellular ATP can be quantitatively detected by measuring the bioluminescence of luciferase. The 4T1 cells were seeded in 24-well plates and then treated with free CPT, P-CPT, and P-DEA-CPT (equivalent CPT dose, 20 μg·mL^−1^) for 24 h. Afterward, cells were harvested and treated according to the standard protocol of the ATP assay kit.

#### 2.6.4. Detection of Caspase 9 and Caspase 3

To investigate the initiation of mitochondrial-related apoptosis pathway, the levels of caspase 9 and caspase 3 were detected. The 4T1 cells were treated with free CPT, P-CPT, and P-DEA-CPT (equivalent CPT dose, 20 μg mL^−1^) for 24 h. Afterward, cells were harvested and treated according to the manufacturer’s illustration of Caspase 9 Activity Assay Kit and Caspase 3 Activity Assay Kit.

### 2.7. Cell Proliferation Suppression Efficacy of HPMA Copolymer–Camptothecin Conjugates

The cell proliferation suppression efficacy of CPT polymeric conjugates was investigated by MTT assay and cell apoptosis assay. For the MTT assay, the 4T1 cells were seeded into a 96-well plate (3 × 10^3^ cells per well) and incubated with free CPT, P-CPT, or P-DEA-CPT at various predetermined concentrations for 48 h. Then, cell viability was determined by MTT reagent. For the apoptosis assay, the 4T1 cells were incubated with CPT, P-CPT, or P-DEA-CPT (equivalent CPT dose, 20 μg mL^−1^) for 24 h. Afterward, the cells were collected and used for apoptosis analysis according to the standard protocols of the Annexin V-FITC/7-AAD Apoptosis Detection kit (BioLegend, San Diego, CA, USA).

### 2.8. In Vitro Anti-Metastasis Assay

The in vitro anti-metastasis effect of CPT polymeric conjugates was evaluated via a migration assay, wound healing assay, and invasion assay. For the migration assay, the 4T1 cells (1 × 10^5^) were suspended in 200 μL of serum-free RPMI 1640 medium and seeded into the upper chamber of transwell inserts. After 4 h incubation, the upper medium was replaced with free CPT, P-CPT, or P-DEA-CPT (equivalent CPT dose, 4 μg∙mL^−1^) and incubated for another 24 h. Subsequently, transwell inserts were fixed with 4% paraformaldehyde followed by staining with crystal violet solution (0.1%). The cells that remained in the upper chamber were wiped out, and the cells that migrated to the lower membranes were imaged under the microscope (Leica Microsystems, Wetzlar, Germany) and dissolved with 33% acetic acid aqueous solution for quantitative determination by Varioskan Flash at 590 nm (Thermo Scientific Varioskan Flash, Waltham, MA, USA).

For the wound healing assay, 4T1 cells were seeded in 24-well plates to grow into a monolayer and scratched with a sterile pipette. Then, cells were incubated with different drug solutions for 24 h. The widths of the wounds at 0 h and 24 h were recorded under microscopy at the same scratched location, and the distance migrated was calculated using Image J software. Furthermore, Matrigel (BD Biosciences, San Diego, CA, USA) was added into the inner bottom of the chamber for 4 h ahead of 4T1 cell seeding, and the invasion assay was performed corresponding to the migration assay.

### 2.9. Intratumoral Mitochondria Targeting Assay

To establish the orthotopic breast cancer mice model, the 4T1 cells (4 × 10^5^ cells) were carefully inoculated into one mammary fat pad of female BALB/c mice. When the tumor size reached about 200–300 mm^3^, mice were intratumorally administrated with free CPT, P-CPT, or P-DEA-CPT (equivalent CPT dose, 5 mg∙kg^−1^). Then, 12 h post injection, tumor tissues were dissected and cut into small pieces followed by trypsin digestion for 15 min. Subsequently, mitochondria in tumor cells were collected according to the standard protocol of Tissue Mitochondria Isolation Kit. A fraction of the collected mitochondria were stained with Mito-Tracker Red (100 nM) for 45 min at 37 °C and visualized under CLSM. Finally, the total fluorescence intensity of CPT in the rest of mitochondria (TFL) was determined via Varioskan Flash and the amount of mitochondrial protein was detected using the Bradford Protein Assay Kit (Keygen Biotechnology, Nanjing, China). The mitochondria accumulation in each group was calculated as the TFL divided by the concentration of mitochondrial protein.

### 2.10. In Vivo Antitumor and Anti-Metastasis Evaluation

When the tumor volume reached about 100 mm^3^ (defined as day 0), orthotopic breast tumor-bearing mice were randomly divided into four groups (*n* = 5) and intratumorally administered saline, free CPT, P-CPT, or P-DEA-CPT (equivalent CPT dose of 5 mg∙kg^−1^, 25 μL) every 3 days for a total of five times. Tumor volumes of mice were detected using a vernier caliper, and the changes in body weight were recorded every 2 days. Tumor volumes were calculated as the following formula: tumor volume (mm^3^) = width^2^ × length/2. For the evaluation of the anti-metastasis effect, mice were sacrificed on day 18, and the excised lungs were immersed in Bouin’s Fluid overnight to count the number of pulmonary metastatic nodules. Subsequently, tumors and the excised organs were fixed in 4% paraformaldehyde for at least 48 h and embedded in paraffin for hematoxylin and eosin (H&E) staining and immunohistochemistry analysis of matrix metalloproteinase-2 (MMP-2), hypoxia inducible factor-1α (HIF-1α), and vascular endothelial growth factor (VEFG).

### 2.11. Statistical Analysis

Student’s *t*-test and one-way analysis of variance (ANOVA) were used to test the significant difference between two groups or multiple groups via SPSS 19.0 software (SPSS, Chicago, IL, USA). Data were expressed as the mean ± standard deviation. Statistical significance was implied using asterisks (* *p* < 0.05, ** *p* < 0.01, *** *p* < 0.001).

## 3. Results and discussion

### 3.1. DEA Content-Dependent Mitochondrial Targeting Capacity of HPMA Conjugates

**Figure 1:**
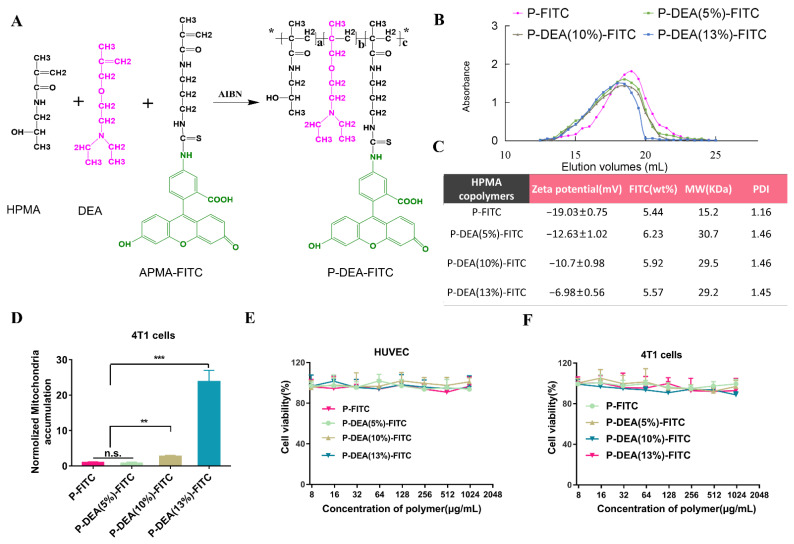
Characterization, mitochondrial targeting capacity, and cytotoxicity of HPMA polymers modified with variable DEA content. (**A**) Graphic illustration of the synthesis route of FITC-labeled HPMA polymers decorated with different proportions of DEA. (**B**) The fast protein liquid chromatography (FPLC) curves and (**C**) the detailed characterizations of FITC-labeled HPMA polymers. (**D**) Mitochondrial accumulation of HPMA polymers with different content of DEA; mean ± SD (*n* = 3). Cell viability of HPMA polymers with different modification degrees of DEA in (**E**) HUVECs and (**F**) 4T1 cells; * *p* < 0.05, ** *p* < 0.01, *** *p* < 0.001, n.s. (no significance); mean ± SD (*n* = 5).

To guarantee efficient mitochondria-targeting capacity, we prepared FITC-labeled HPMA polymers with various proportions of DEA decorated on the side-chains to select an optimal amount of DEA. FITC-labeled HPMA polymers containing 0–13% molar ratio of DEA (P-FITC, P-DEA (5%)-FITC, P-DEA (10%)-FITC, and P-DEA (13%)-FITC) were prepared via one-step radical homo-polymerization according to our previous reports [25] (Figure 1A). As depicted in Figure 1B,C, the molecular weights of these copolymers were in the range of 15.2 to 29.5 kDa, and the PDIs were in the range of 1.16 to 1.46. The FITC loading (5.44–6.23 wt.%) of each group was at a similar level. Furthermore, the gradually increased zeta potentials of these polymers revealed the different modification degrees of the positively charged DEA (Figure 1C). Notably, the DEA content-dependent mitochondria-targeting capacity was observed, and P-DEA (13%)-FITC mediated approximately 24-fold higher mitochondrial accumulation than P-FITC (Figure 1D). In addition, the cell viabilities of HUVECs and 4T1 cells were both over 90% at concentrations of P-DEA (13%)-FITC up to 1000 μg·mL^−1^ (Figure 1E,F). These results strongly verified that the 13% molar proportion of DEA was optimal to induce efficient mitochondria location of HPMA polymers with good cytocompatibility.

### 3.2. Synthesis and Characterization of HPMA Copolymer–CPT Conjugates

**Figure 2:**
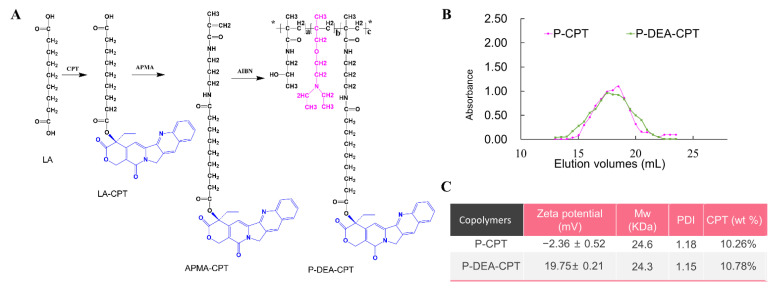
Synthesis and characterization of HPMA copolymer–CPT conjugates. (**A**) Schematic representation of the synthesis route of P-DEA-CPT. (**B**) The fast protein liquid chromatography (FPLC) curves and (**C**) detailed characterizations of P-CPT and P-DEA-CPT; * *p* < 0.05; mean ± SD (*n* = 3).

HPMA copolymer–CPT conjugates were synthesized as shown in Figure 2A. Briefly, CPT polymeric conjugate decorated with 13 mol.% of DEA (P-DEA-CPT) was synthesized via direct radical polymerization of HPMA, DEA, and APMA–CPT and initiation of azobisisobutyronitrile. Furthermore, a CPT polymeric conjugate without DEA modification (P-CPT) was prepared as a control. As displayed in Figure 2B,C, the molecular weight of P-DEA-CPT was 23.3 kDa with a PDI of around 1.1. P-DEA-CPT displayed a successful CPT loading of ~10 wt.%. Similar properties were also observed in P-CPT. Moreover, zeta potentials revealed that P-DEA-CPT with DEA modification had a significantly stronger positive charge as compared with P-CPT.

### 3.3. Cellular Uptake, Lysosome Escape, and Mitochondrial Targeting of P-DEA-CPT

**Figure 3:**
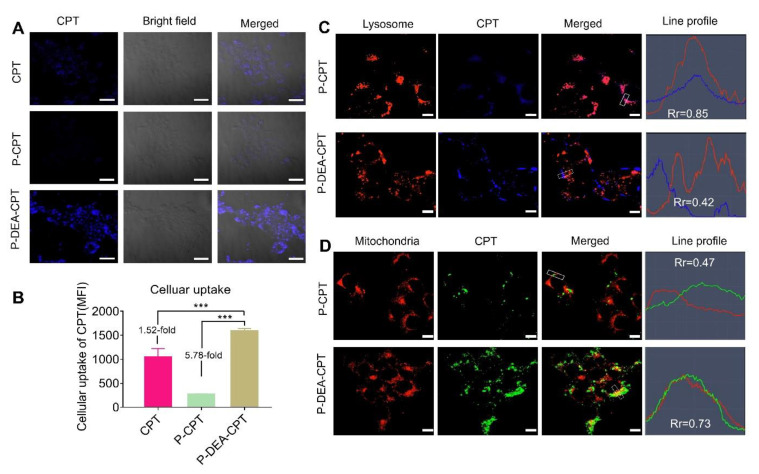
Subcellular trafficking of HPMA copolymer–CPT conjugates in cancer cells. (**A**) Qualitative CLSM and (**B**) quantitative flow cytometry analysis of the cellular uptake in 4T1 cells. Scale bar: 20 μm; mean ± SD (*n* = 3). (**C**) Lysosome escape and (**D**) mitochondria localization of HPMA copolymer–CPT conjugates in 4T1 cells observed under CLSM. Scale bar: 10 μm. *** *p* < 0.001; mean ± SD (*n* = 3).

To investigate whether DEA could facilitate internalization of HPMA copolymer-CPT conjugates in cancer cells, cellular uptake of P-DEA-CPT was investigated in 4T1 cells. CLSM observation displayed that the fluorescence intensity of FITC was significantly stronger in the P-DEA-CPT group than that in the P-CPT group (Figure 3A). Consistently, quantitative analysis by flow cytometry indicated a 5.7-fold higher internalization of P-DEA-CPT over P-CPT (Figure 3B). After entering cancer cells, P-DEA-CPT was expected to escape from lysosome and accumulate in mitochondria. To verify this, the subcellular trafficking of the internalized polymers was examined. The results displayed that most of the P-CPT was trapped in the lysosome (Rr = 0.85, between CPT and lysosome, Figure 3C), whereas a small proportion of P-CPT was localized in the mitochondria (Rr = 0.55, between CPT and mitochondria, Figure 3D). Encouragingly, P-DEA-CPT could largely escape from lysosome (Rr = 0.44, between CPT and lysosome, Figure 3C) and subsequently accumulate in the mitochondria (Rr = 0.70, between CPT and mitochondria, Figure 3D). Thus, the modification of DEA not only improved the internalization of copolymer conjugates but also facilitated lysosome escape and mitochondrial colocalization.

### 3.4. In Vitro Mitochondrial Damage-Induced Apoptosis and Cytotoxicity of P-DEA-CPT

**Figure 4:**
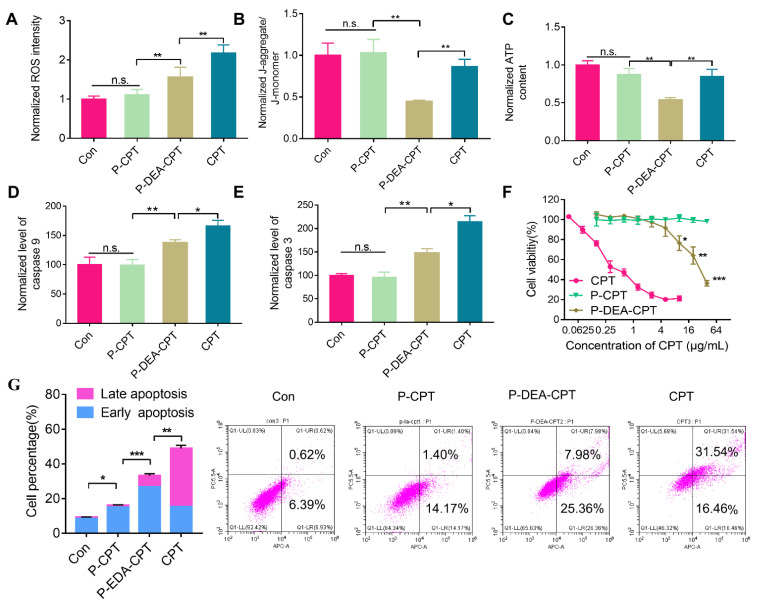
P-DEA-CPT damaged the mitochondria, initiated the mitochondrial apoptosis pathway, and caused cancer cell death. (**A**) Intracellular ROS level, (**B**) mitochondrial membrane potential depolarization, (**C**) ATP generation, (**D**) caspase-9 expression, and (**E**) caspase-3 expression in 4T1 cells were determined after different treatments. (**F**) Cell viability of 4T1 cells after being treated with different CPT polymers for 48 h. (**G**) The mean value and representative images of apoptotic and necrotic 4T1 cells induced by CPT polymers, as tested by flow cytometry. * *p* < 0.05, ** *p* < 0.01, *** *p* < 0.001, n.s. (no significance); mean ± SD (*n* = 3).

Mitochondria perform pivotal roles in determining cancer cell fate. The damage can directly cut down the energy supply and activate a variety of key events to initiate intrinsic pathways of apoptosis [26,27]. We investigated whether the enhanced delivery of CPT to mitochondria could result in mitochondria dysfunction. Notably, a significant increase in ROS was observed in the P-DEA-CPT group compared with P-CPT (Figure 4A). Once CPT inhibited the mitochondrial respiratory chain complexes, the electron transfer in the electron transfer chain would be blocked and unstable oxygen would leak out from mitochondria, subsequently forming ROS [28,29]. Thus, the overload of intracellular ROS induced by P-DEA-CPT revealed the impaired mitochondrial function by CPT. Moreover, mitochondrial membrane potential depolarization is another sign of mitochondrial dysfunction. JC-1 is a cationic lipophilic probe with Δψm-dependent aggregation in the mitochondria, and the transition from the red J-aggregate to the green J-monomer is a direct measurement of the decreased mitochondrial membrane potential. As displayed in Figure 4B, P-DEA-CPT caused a sharper drop in the red (aggregate)/green (monomer) fluorescence intensity ratio than both free CPT and P-CPT, indicating the significant Δψm loss (52.6% decrease compared to control). Furthermore, the majority of ATP in tumor cells is produced by the mitochondria aerobic respiration. Therefore, the dysfunction of mitochondria would inevitably reduce ATP production. As shown in Figure 4C, P-DEA-CPT exhibited maximum ATP decline in the 4T1 cells among all the groups, suggesting the most severe mitochondrial dysfunction.

It has been reported that mitochondria play crucial roles in regulating the intrinsic apoptotic pathways of cancer cells [30]. Upon inducing mitochondrial apoptosis, caspase 9 and caspase 3 would be activated, subsequently amplifying the apoptotic cascade [31]. Thus, caspase 9 and caspase 3 were determined to investigate whether mitochondrial dysfunction could activate the mitochondrial apoptotic pathway. After being incubated with P-DEA-CPT for 24 h, the expressions of caspase 9 and caspase 3 in 4T1 cells were both significantly upregulated as compared with P-CPT (Figure 4D,E). Consequently, P-DEA-CPT induced significantly stronger cytotoxicity (Figure 4F) with much higher cell apoptosis than P-CPT (Figure 4G). DNA-damaging drugs that cause apoptosis also rely on the initiation of caspase proteins [32,33]. Thus, free CPT-induced dose-dependent cytotoxicity was ascribed to the direct destruction of nuclear DNA, resulting in the elevated release of caspase 9 and caspase 3. Taken together, by impairing mitochondrial function, P-DEA-CPT efficiently blocked the energy generation and curbed proliferation of cancer cells, ultimately potentiating the therapeutic outcomes in cancer therapy.

### 3.5. In Vitro Anti-Metastasis Effect of P-DEA-CPT

**Figure 5:**
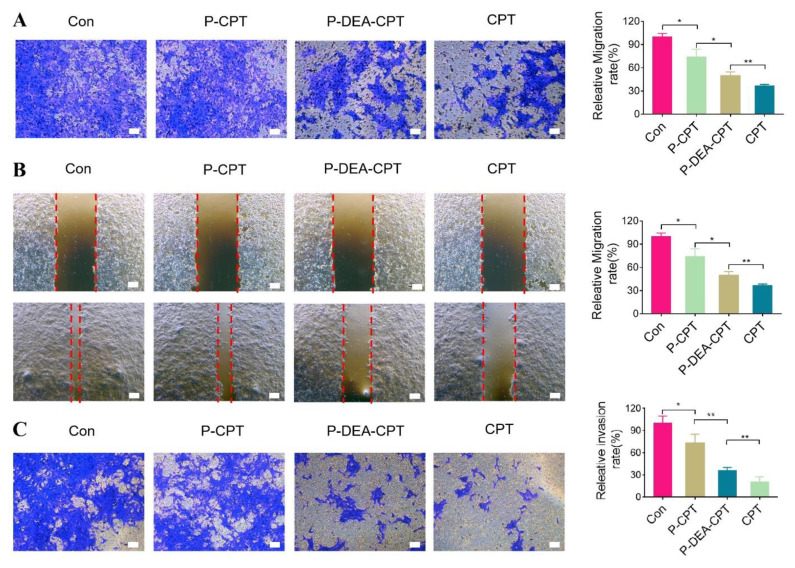
Anti-metastasis effect of P-DEA-CPT in vitro. (**A**) Migration, (**B**) wound healing, and (**C**) invasion of 4T1 cell lines after being treated with free CPT, P-CPT, and P-DEA-CPT for 24 h, as displayed via representative images and quantitative results. Migrant or invasive cells (at the outer bottom of the transwell chamber) were stained with crystal violet and measured by the microplate reader. Scale bar: 200 μm. * *p* < 0.05, ** *p* < 0.01; mean ± SD (*n* = 3).

Cancer metastasis involves complex cell biological cascades. Tumor cells migrate into the surrounding tumor stroma and invade through basement membranes, followed by entering blood circulation [34,35,36]. Thus, whether this mitochondria-targeting strategy could impede the processes of tumor-metastasis was investigated. As shown in Figure 5A, P-CPT only displayed a slight migration inhibitory effect (~75% of migration rate). In stark contrast, a much lower migration rate (~50%) was observed in P-DEA-CPT group. The wound healing assay also displayed that P-DEA-CPT exhibited a significantly stronger inhibitory effect on the mobility of 4T1 cells than P-CPT (52.9% vs. 25.5%, Figure 5B). Furthermore, P-DEA-CPT induced potent invasion restraint of 4T1 cells, and the invasion rate decreased to 36.45% (Figure 5C). Although free CPT inhibited the migration and invasion of 4T1 cells due to its certain cytotoxicity and anti-proliferation capacity, it might also cause unexpected side-effects. These results validated that P-DEA-CPT could potently suppress the migration, mobility, and invasion of 4T1 cells, providing great promise for inhibiting cancer cells from disseminating into circulation.

### 3.6. Intratumoral Mitochondrial Targeting of P-DEA-CPT

**Figure 6:**
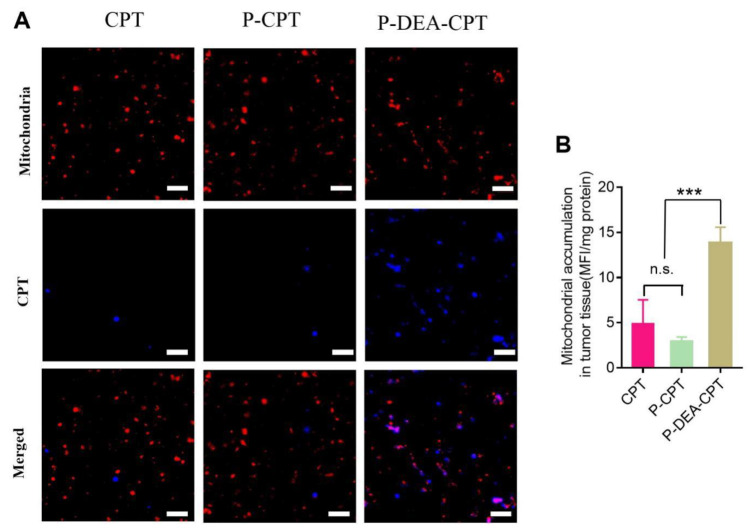
Intratumoral mitochondrial targeting of P-DEA-CPT in 4T1 orthotopic breast tumor. Mitochondria-specific colocalization of HPMA copolymer conjugates and free CPT in 4T1 cells as (**A**) observed under CLSM and (**B**) quantitatively detected via microplate reader. Red, mitochondria; blue, CPT. Scale bar: 10 μm. *** *p* < 0.001, n.s. (no significance); mean ± SD (*n* = 3).

Due to the cationic feature afforded by DEA modification on the side-chain, the polymer P-DEA-CPT could achieve enhanced internalization into tumor cells due to the affinity to the negatively charged tumor cell membrane upon intratumoral injection [37]. Then, P-DEA-CPT could achieve lysosome escape due to the “proton sponge effect” [38]. Lastly, according to the negative nature of mitochondrial inner membrane (∆Ψ = −150 to −180 mV), P-DEA-CPT could effectively target mitochondria in tumor cells [39]. To verify this, whether P-DEA-CPT could locate in mitochondria within tumor tissues was then further evaluated. Intratumoral mitochondrial targeting experiments displayed that P-CPT exhibited negligible mitochondria colocation under CLSM observation, whereas the strong dotted blue signals of P-DEA-CPT overlapped well with the red signals of mitochondria (Figure 6A). Consistently, quantitative analysis revealed a 4.7-fold higher mitochondrial accumulation of P-DEA-CPT as compared with P-CPT (Figure 6B). These results confirmed that P-DEA-CPT could successfully accumulate in the mitochondria within tumor tissues, laying the foundations for in vivo treatment.

### 3.7. In Vivo Antitumor and Anti-Metastasis Effect of P-DEA-CPT

**Figure 7:**
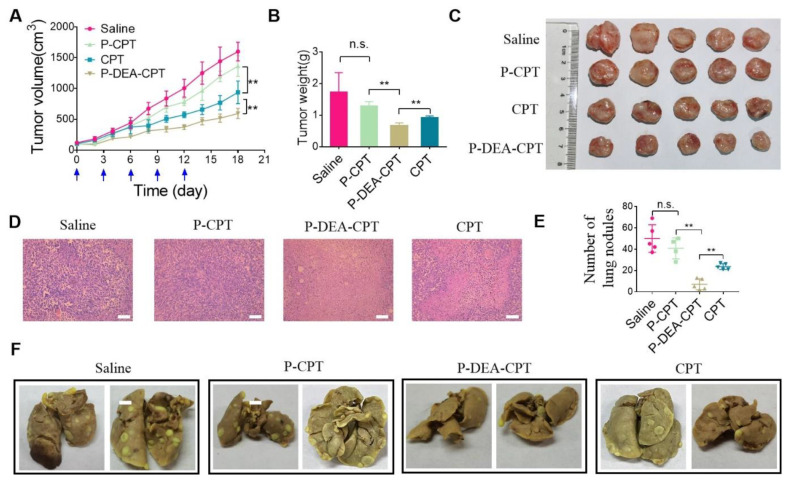
In vivo antitumor and anti-metastasis effect of P-DEA-CPT in 4T1 orthotopic breast tumor. (**A**) Average tumor growth curves, (**B**) tumor weight at the end of the assay, (**C**) representative images of the excised tumor tissues, and (**D**) H&E results of tumor sections in each treatment group. Scale bar: 200 μm. (**E**) The number of metastatic nodules and (**F**) representative images of excised lung stained by Bouin’s fluid in each treatment group. ** *p* < 0.01, n.s. (no significance); mean ± SD (*n* = 5).

**Figure 8:**
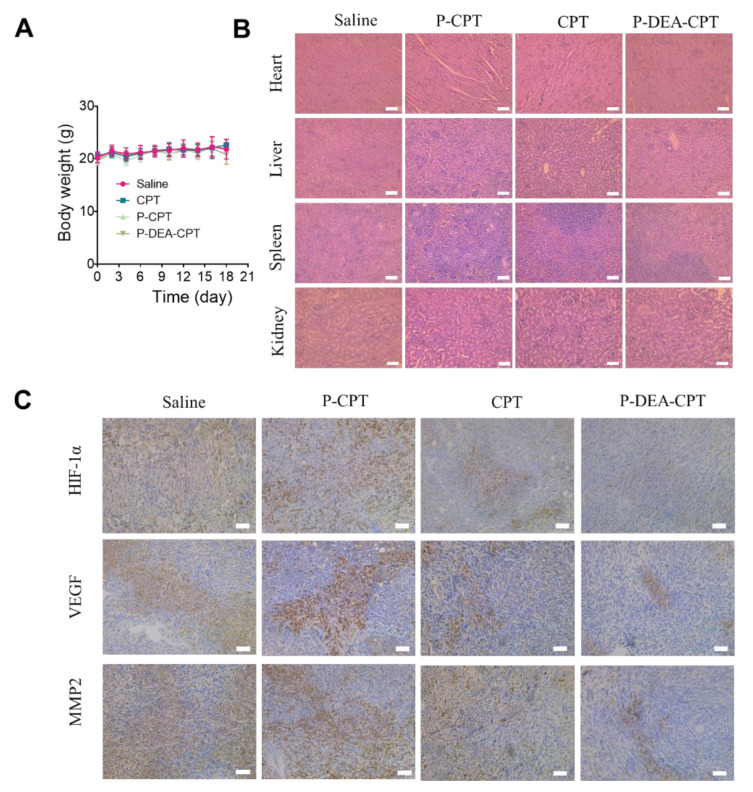
(**A**) Body weight changes and (**B**) H&E staining of major organs of all the groups during the treatment period in 4T1 orthotopic breast tumor. (**C**) Immunohistochemistry staining of tumor sections for analysis of the expression of HIF-1α, MMP-2, and VEGF at the endpoint. The brown area indicates the positive expression of these proteins. Scale bar: 200 μm.

Triple-negative breast cancer (TNBC) orthotopic murine models can simulate multiple phases of metastasis formation [40,41]. Motivated by the potent anti-metastasis capacity in vitro, the orthotopic 4T1 breast tumor model was established as the method mentioned above to evaluate the in vivo antitumor and anti-metastasis effect of P-DEA-CPT. When tumor volume reached around 100 cm^3^, free CPT, P-CPT, and P-DEA-CPT (equivalent CPT dose of 5 mg·kg^−1^, 25 μL) were intratumorally injected every 3 days for a total of five times. At the end of the treatment, P-CPT displayed a negligible tumor growth-inhibitory effect. In sharp contrast, P-DEA-CPT potently curbed tumor growth (inhibition rate of 61.2%) with the slowest tumor growth rate and lowest tumor weight among all the groups (Figure 7A–C). It is also worth noting that free CPT only exhibited a moderate antitumor effect despite its much stronger cytotoxicity than P-DEA-CPT in vitro. This might be attributed to its rapid clearance in tumor tissues via vascular leakage, whereas the high-molecular-weight polymer remained in tumor tissue for a longer time [42,43]. Hematoxylin and eosin (H&E) staining results further validated the best tumor growth suppression capacity of P-DEA-CPT with a larger area of apoptosis (Figure 7D). At the end of the assay, the excised lungs were immersed in Bouin’s fluid, and the metastatic nodules were counted to evaluate the lung metastasis formation in each treatment group. Obviously, mice in the saline group developed serious lung metastasis, and an imperceptible metastasis suppression effect was observed in both P-CPT and free CPT groups (Figure 7E,F). Comparably, P-DEA-CPT greatly decreased lung metastasis with the fewest lung nodules among all the groups (Figure 7E,F). In addition, all the groups exhibited favorable safety with no weight loss and histopathological changes in major organs during the treatment period (Figure 8A,B). These results manifested that P-DEA-CPT attained the efficient anti-tumor and anti-metastasis capacity with considerable safety.

The potent anti-metastasis effect of P-DEA-CPT could be partially ascribed to its energy depletion and enhanced apoptosis caused by impairing mitochondria. Moreover, mitochondria dysfunction was reported to alleviate hypoxia and downregulate hypoxia-inducible factor (HIF-1α) [44,45], the key regulator for various pro-metastasis downstream signaling pathways, resulting in the expressions of vascular endothelial growth factor (VEGF) and matrix metalloproteinases (MMPs) [46,47]. To verify this, the expression of crucial factors for tumor metastasis (HIF-1α, MMP2, and VEGF) was investigated via immunohistochemistry assay. As shown in Figure 8C, remarkably downregulated expressions (lighter brown) of HIF-1α, MMP-2, and VEGF were observed in P-DEA-CPT group. Collectively, the great efficacy of our mitochondrial targeting strategy to inhibit tumorigenesis and metastasis largely originates from multiple mechanisms, including cutting down energy supply, activating the intrinsic apoptotic pathway, and downregulating HIF-1α and its downstream pro-metastasis factors (MMP2 and VEGF).

## 4. Conclusions

In summary, we fabricated a mitochondria-targeting CPT delivery platform based on water-soluble HPMA copolymers with an optimal content of DEA modified on the side-chain. Firstly, the water-soluble DEA sufficiently escorted the CPT conjugates located in the mitochondria both in 4T1 cells and in tumor tissues. Secondly, P-DEA-CPT elicited serious mitochondrial dysfunction with the decline in ATP and activation of proapoptotic caspase proteins, thereby resulting in the restraint of cancer cell proliferation, migration, and invasion. Furthermore, the expressions of HIF-1α and its downstream metastasis-associated proteins including MMP-2 and VEGF in tumor sites were notably inhibited. Consequently, both primary breast cancers and distant pulmonary metastases were largely impeded. Overall, our study revealed that DEA is a promising candidate to escort CPT to accumulate in the mitochondria. Furthermore, selectively delivering CPT to the mitochondria provides a feasible way of improving the anti-metastasis effect.

## Data Availability

Not applicable.
